# Prolonged outbreak of *Serratia marcescens* in Tartu University Hospital: a case–control study

**DOI:** 10.1186/1471-2334-12-281

**Published:** 2012-10-31

**Authors:** Vivika Adamson, Piret Mitt, Heti Pisarev, Tuuli Metsvaht, Kaidi Telling, Paul Naaber, Matti Maimets

**Affiliations:** 1 Department of Infection Control, Tartu University Hospital, Puusepa 1a, 50406, Tartu, Estonia; 2 Department of Public Health, University of Tartu, Tartu, Estonia; 3 Pediatric Intensive Care Unit, Intensive Care Clinic, Tartu University Hospital, Tartu, Estonia; 4 Department of Medical Microbiology, Stavanger University Hospital, Stavanger, Norway; 5 Department of Internal Medicine, Tartu University Hospital, Tartu, Estonia

## Abstract

**Background:**

The aim of our study was to investigate and control an outbreak and identify risk factors for colonization and infection with *Serratia marcescens* in two departments in Tartu University Hospital.

**Methods:**

The retrospective case–control study was conducted from July 2005 to December 2006. Molecular typing by pulsed field gel electrophoresis was used to confirm the relatedness of *Serratia marcescens* strains. Samples from the environment and from the hands of personnel were cultured.

**Results:**

The outbreak involved 210 patients, 61 (29%) developed an infection, among them 16 were invasive infections. Multivariate analysis identified gestational age, arterial catheter use and antibiotic treatment as independent risk factors for colonization and infection with *Serratia marcescens.* Molecular typing was performed on 83 *Serratia marcescens* strains, 81 of them were identical and 2 strains were different.

**Conclusions:**

Given the occasionally severe consequences of *Serratia marcescens* in infants**,** early implementation of aggressive infection control measures involving patients and mothers as well as the personnel is of utmost importance.

## Background

Neonates admitted to neonatal intensive care units (NICUs) are at high risk of nosocomial infection (NI) [1-3]. NIs due to gram-negative rods are usually caused by bacterial strains that have already colonized the infants [4]. Most neonates acquire *Enterobacteriaceae*, primarily different strains of *Klebsiella sp*, *E. coli* and *Enterobacter sp. Serratia sp.* is a gram-negative rod-shaped bacterium that may colonize the nasopharynx and the gut and occasionally causes invasive (bloodstream infection, meningitis, pneumonia) and noninvasive (urinary tract, conjunctivitis) infections in neonates. During the last decade several articles have been published about outbreaks caused by *Serratia sp.,* especially in neonatal intensive care units [5-9]. Independent risk factors for colonization and infection with *Serratia sp.* are not well established, but low birth weight, prematurity, prolonged hospital stay, antibiotic use and mother’s infections prior to delivery have been found to be risk factors according to some investigators [5,6,9,10]. The aim of our study was to describe the outbreak and identify risk factors for colonization and infection with *Serratia marcescens* in the Pediatric Intensive Care Unit (PICU) and the Neonatal Unit (NU) in Tartu University Hospital from July 2005 to December 2009.

## Methods

### Setting

Tartu University Hospital is a 960-bed tertiary care teaching hospital with approximately 43 000 admissions annually. The study was conducted in two units: a 9-bed PICU and a 24-bed NU, which serve 300 and 800 admissions per year, respectively. Approximately 2/3 of PICU patients are neonates and 1/3 older children. Neonates, who no longer require intensive care are transferred to NU on the same floor. If the condition should worsen, neonates can be transferred back to PICU.

Neonatologists and trained neonatal nurses work in the units. The nurse to patient ratio is usually up to 1:2 in PICU, in NU there is a separate nurse for patients requiring intermediate care (6 beds) and two nurses for the other patients.

The antibiotic policy at the time of our study recommended benzylpenicillin/ampicillin and gentamicin as the first-line empirical treatment for suspected early-onset sepsis. This study was approved by Research Ethics Committee of the University of Tartu (UT REC), 215/T-8.

### Microbiological methods

In order to investigate the outbreak samples for surveillance, cultures were obtained from nares and rectum of all patients on admission and twice a week as part of standard care in both units. Nasal and rectal swabs were plated on agar (DNAase Agar Test Agar with Methyl Green, Becton Dickinson, USA) selective for *Serratia* species.

Isolates of *Serratia marcescens* from clinical or screening specimens were identified by using the identification method routinely employed in our clinical microbiology laboratory and verified by biochemical identification system VITEK2 Compact (BioMerieux, France) using GN card. Antimicrobial susceptibility was determined according to the recommendations of the Clinical and Laboratory Standards Institute [11]. All isolates were stored at −80°C for future analysis.

Environmental cultures were obtained from potential sources such as sinks, soaps, different surfaces (incubators, perfusors), medical equipment (oxygen tubes, oxygen masks, feeding syringes, breast pumps) and opened vials and bottles (antibiotics, saline and glucose solutions). In addition, hands of the personnel were cultured twice. Pre moistened swabs (APTACA, Amies transport medium, Italy) were used for obtaining cultures from the environment as well as from the hands of health care workers. The specimens were plated and evaluated in the same manner as the patients’ surveillance cultures.

### Typing

Molecular analysis of each isolate was performed by pulsed field gel electrophoresis (PFGE). DNA plugs were prepared by following manufacturer’s instructions for the GenePath Group 3 Reagent kit (Bio-Rad Laboratories, Hercules, CA, USA). After restriction enzyme digestion with 25 U of *SpeI*, gels were electrophoresed for 22 h at 14°C at a constant voltage 6V/cm using the CHEF-DR III system (Bio-Rad). Isolates were considered to be the same genotype using the criteria described by Tenover et al [12].

### Case–control study

**Figure 1:**
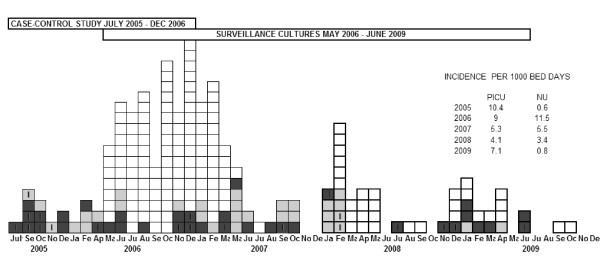
**Epidmic curve for an outbreak with *Serratia marcescens *in Tartu University Hospital.** (white box - colonization, black box - infection in unit A, gray box - infection in unit B, I - invasive infection).

To identify risk factors for colonization or infection with *Serratia marcescens* we conducted retrospective case–control study among patients hospitalized from July 2005 to December 2006 (Figure 1).

A case was defined as a neonate, who was admitted to PICU or NU during the period from July 2005 to December 2006 with at least one clinical or surveillance culture positive for *Serratia marcescens*. Neonates whose cultures remained negative for *Serratia marcescens* served as controls. Children older than 28 days were not included in the case–control study.

Colonization was defined as a positive culture for *Serratia marcescens* in the absence of infection symptoms. Infection was defined as presence of infection symptoms simultaneously with isolation of *Serratia marcescens* from a clinical specimen.

Risk factors were evaluated retrospectively from medical records including demographic characteristics, gestational age, birth weight, Apgar scores, duration of mechanical ventilation, exposure to indwelling devices, nutrition (parenteral and enteral feeding), surgery and antimicrobial therapy. Finally, risk factors for colonization and infection were analyzed separately.

### Statistical analysis

Logistic regression analysis was used to calculate odd ratios and 95% confidence limits to describe differences in risk factors. Significant factors from univariate analysis were used to perform multivariate analysis. Statistical significance was set at p<0.05.

The statistical analyses of the data were performed using the statistical software package R 2.4.0 - A Language and Environment.

## Results

### Outbreak description

The outbreak investigation was conducted in two departments after *Serratia marcescens* was isolated from blood cultures of three patients in short intervals. The retrospective analysis of the microbiology laboratory database showed that *Serratia marcescens* colonization or infection had not been detected in neither of the departments during two previous years (2003 and 2004).

From July 2005 to December 2009 a total of 210 patients with *Serratia marcescens* colonization or infection were identified. At the end of 2006 and in the beginning of 2007 the number of cases reached the maximum and began to decrease in 2008.

In 2010 there have been no cases. The distribution of cases by months and exact numbers per 1000 bed days are presented on an epidemic curve (Figure 1).

Out of 210 patients 111 (53%) were males and 99 (47%) females. Most of them (n=181) were neonates, 27 children were under the age of 1 year and 2 children were older than 1 year. Among all neonates (n=181) 27 (15%) were born at term and 154 (85%) were preterm (birth before 37 weeks of gestation). Among preterm neonates 67 had a birth weight <1500 g and 22 weighed <1000 g.

Out of 210 patients 61 (29%) developed an infection: 16 of these were invasive infections (bloodstream infection, meningitis). 149 of these patients were colonized, most of them at more than one site.

The mean length of stay in PICU until the first positive culture was 12.4 (range 1 to 64) days and in NU 11.8 (range 1 to 74) days.

### Case–control Study

The case–control study included 105 case-patients and 206 control-patients. Risk factors associated with acquisition of *Serratia marcescens* in univariate analysis are presented in Table 1. Multivariate analysis with logistic regression was performed to control confounding variables. Multivariate analysis identified gestational age, arterial catheter use and antibiotic treatment as independent risk factors for colonization and infection with *Serratia marcescens* (Table 2).

**Table 1:** **Univariate anaysis of risk factors associated with *Serratia marcescens *infection or colonzation**

**Characteristic**	**Case patients (n= 105)**	**Control patients (n= 206)**	**OR**	**CI95**
Mean gestational age, wk	34	37	0,76*	0,69–0,83
(range; OR per week)	(23–41)	(25–42)		
Mean birth weight, g	2235	2986		
(range; OR per 500g)	(500–5220)	(400–5850)	0,56*	0.6–0.68
Vaginal delivery	50 (48%)	132 (64%)		
Cesarean section	55 (52%)	74 (36%)	1,92*	1.18–3.12
Apgar score after 1 min	6	7	0,84*	0.75–0.93
Prematurity	75 (71%)	74(36%)	5.46*	3.01–9.93
Invasive device				
Intubation	35 (33%)	27 (13%)	2,18*	1,55–8,43
Mean duration of intubation, d	8,1	3,7		
(range)	(1–61)	(1–24)	1,06	0,93–1,22
CVC	31 (30%)	31 (15%)	2,87*	1,51–5,46
Mean duration of CVC,d	10	7,4		
(range)	(1–42)	(1–30)	1,08*	1,02–1,13
Arterial catheter	51 (49%)	45 (22%)	10,44*	4,06–26,83
Peripherial catheter	102 (97%)	160 (78%)	10,18*	3,09–33,53
Nasogastral tube	86 (82%)	88 (43%)	6,86*	3,57–10,07
Parenteral feeding	28 (27%)	25 (12%)	4,72*	2,27–9,8
Breast milk	77 (73%)	179 (87%)	0,37*	0,19–0,71
Antimicrobial therapy	93 (89%)	100 (51%)	10,86*	4,63–25,46
Mean duration of antimicrobial therapy, d	7,5	6,5	1,03	0,98–1,08
(range)	(1–52)	(1–29)		
Maternal infection	21 (20%)	45 (24%)	0,84	0,46–1,53
Mean length of stay, d	31	13	1,05*	1,03–1,07
(range)	(3–213)	(2–90)		
Surgery	14 (13%)	11 (5%)	2,72*	1,16–6,37
Outcome (death)	4 (4%)	3 (2%)	3,61	0,65–19,91

Note. Time-dependent risk factors have been evaluated until the first positive culture for *Serratia marcescens.*

*OR* odds ratio, *CI95* 95% confidence interval.

*statistical significance (p< 0.05).

**Table 2:** **Multivariate analysis of factors associated with *Serratia marcescens *colonization or infection**

**Variable**	**OR**	**CI95%**
Gestational age	0,82	0,75–0,91
Arterial catheter	4,49	1,57–12,82
Antimicrobial therapy	6,01	2,42–14,96

Note. - *OR* odds ratio, *CI95* 95% confidence interval.

The analysis of risk factors for colonization and infection separately showed that statistically significantly more arterial catheters had been used in infected patients (OR 1.67 CI95% 1.16 – 2.4).

### Genotyping and Antibiotic Susceptibility

**Figure 2:**
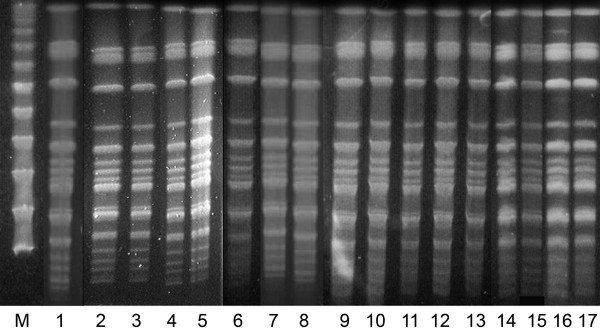
**Pulsed field gel electrophoresis of *Serratia marcescens *strains isolated from infected or colonized patients and environment.** M - lambda marker; lane 1 - environmental culture; lane 2–17 strains from infected or colonized patients.

Molecular typing was performed on 83 *Serratia marcescens* strains, 81 of them were identical (clone A, Figure 2) and 2 strains were different (clone B). All isolates were *in vitro* susceptible to cefotaxime, cefepime, piperacillin/tazobactam, meropenem, imipenem, gentamicin, amikacin and ciprofloxacin. All isolates were resistant to ampicillin, ampicillin/sulbactam and cefuroxime.

### Infection control measures

All cultures (n= 189) obtained from inanimate surfaces in the wards were negative for *Serratia marcescens* with the exception of the 0.9% NaCl syringe, which was used for a child, who had been previously colonized with *Serratia marcescens.*

None of the cultures obtained from personnel hands yielded *Serratia marcescens*.

Attempts were made to separate infected neonates from non-infected neonates. Cohorting the personnel turned out to be a problem due to the lack of health care workers.

In order to improve the availability of hand antiseptics, dispensers of hand antiseptics were placed at all incubators. The personnel was educated to revise the rules of hand hygiene and attention was paid to the compliance with hand hygiene recommendations. The usage of hand antiseptics was calculated on the basis of pharmacy’s database.

According to the recommendations of the infection contol team, disposable feeding syringes were taken into use and breast pumps were made available in every patient room in the NU. The algorithm of cleaning the breast pumps was also intensified. The ward was supplied with an instrument washing machine.

## Discussion and conclusion

*Serratia sp.,* formerly considered to be a benign commensal only, is now recognized as an important cause of outbreaks, especially in neonatal intensive care units 16,22. According to our knowledge this is the largest published outbreak in terms of case numbers and duration.

Our study showed that gestational age, antimicrobial therapy and arterial lines are significantly associated with colonization and infection with *Serratia marcescens.*

The development of the intestinal microflora starts at birth and is influenced by various factors such as gestational age, mode of delivery, local environment, type of feeding and antibiotic treatment [13,14].

The role of antibiotics as a risk factor has been investigated in several similar case–control studies [5,6,9,10]. Statistically significant associations between previous antibiotic treatment of mothers [5,6] and neonates [10] have been found by some authors. It is known that antibiotic treatment may affect the colonization with gram-negative bacteria. Antibiotic therapy suppresses formation of normal flora, reduces remarkably colonization resistance and increases risk of acquiring a nosocomial strain [4]. It is difficult to estimate to what extent antibiotic treatment influenced our outbreak. We suppose that our empirical antibiotic treatment didn`t influence the acquisition of Serratia significantly, the major factor was rather the poor compliance to infection control measures. We did not change the guidelines of empirical antibacterial treatment at a time of the outbreak, because in similar studies its significance in terminating the outbreak has remained unclear [16].

The second finding was the presence of an arterial line as an independent risk factor for colonization and infection with *Serratia marcescens*. Other studies have also shown invasive intravascular devices as risk factors for colonization and infection with *Serratia sp.*[5,17,18]. However, Maragakis et al. [17] suppose that these are rather markers of severity of illness and susceptibility of a patient to infection than independent risk factors. We also agree with this statement and presume that the usage of these devices has probably no direct causal role in colonization with *Serratia marcescens*. On the other hand, invasive device use increases the number of contacts with personnel and thus, in case of suboptimal hand hygiene, increases the possibility of transmission. In order to improve insertion and care of intravascular catheters, including arterial catheters, a guideline was drawn up and implemented.

As in most of the other outbreak studies, the reservoir and mode of transmission remained unknown also in our study. We suppose that asymptomatic patients are the most important reservoir of *Serratia sp.*, because after the implementation of screening policy (included samples from nasopharynx and rectum) and isolation precautions the number of new cases decreased significantly. As in our study the gastrointestinal tracts as well as respiratory tracts of neonates were frequently colonized, we agree with Giles et al. [19] that during *Serratia sp.* outbreak both respiratory and gastrointestinal samples should be collected in order to maximize the identification of colonized infants.

There is a likelihood that the organisms may have been transmitted via the hands of health care workers and mothers. This hypothesis is supported by the evidence that no other cultures taken from the environment were positive. However, cultures taken from the hands of health care workers were negative. In 2001–2003 a study conducted in New York Presbyterian Hospital in New York City aimed to determine the relative frequency of potential horizontal transmission between patients and health care workers in the NICU. This large prospective study indicated a potentially higher probability of cross-transmission with certain gram-negative bacilli, such as *Klebsiella pneumoniae* and *Serratia marcescens* via the hands of health care workers [20]. We speculate that in addition to personnel, mothers may have had a role in transmission of *Serratia sp*. in the ward. Hand hygiene rules were explained to mothers, but their compliance was not followed routinely. Unfortunately no samples were cultured from mothers’ hands during the outbreak.

Closing the ward temporarily has been an efficient measure to terminate an outbreak [6,21]. This option was under discussion in our case, but it was not possible to transfer intensive care patients to another hospital (due to long distance) or to open an additional ward (lack of qualified personnel). It was also impossible to employ additional personnel.

Limitation of our study concerns the aspect that among control patients there could be patients colonized with other bacteria due to the fact that a selective culture for *Serratia sp.* was used for screening*.* It is possible that previous colonization with other gram-negative bacteria causes colonization resistance of the gastrointestinal tract and reduces the opportunity to colonize with potentially pathogenic microorganisms such as *Serratia marcescens*. On the other hand, as the length of stay in PICU was 12.4 days till the first positive culture and in NU 11.8 days, there is a possibility that case-patients were colonized with other gram-negative bacteria also by that time.

In conclusion, we can say that as the implementation of infection control measures was delayed, the outbreak lasted a long time. In order to terminate an outbreak caused by *Serratia sp.* promptly, early implementation of thoroughly considered aggressive infection control measures involving patients and mothers as well as the personnel is of utmost importance. Continued attention should be paid on optimizing antibiotic usage.

## Competing interests

All authors report no conflicts of interest relevant to this article.

## Authors’ contributions

VA planned the study, carried out the data collection and analysis, performed PFGE of all collected strains and drafted the manuscript. HP participated in the design of the study and performed statistical analysis. PM participated in the design of the study and data analysis and drafted the manuscrip. TM participated in coordination of the study and helped to draft the manuscript. KT participated in the data collection and helped to carry out the molecular studies PN helped to plan and design the study, participated in coordination in the field of microbiology and drafted the manuscript. MM conceived the study and participated in its design and coordination and helped to draft the manuscript. All authors read and approved the final manuscript.

## Pre-publication history

The pre-publication history for this paper can be accessed here:

http://www.biomedcentral.com/1471-2334/12/281/prepub
